# Left Atrial Low-Voltage Areas Predict the Risk of Atrial Fibrillation Recurrence after Radiofrequency Ablation

**DOI:** 10.3390/biomedicines11123261

**Published:** 2023-12-09

**Authors:** Raluca-Elena Mitran, Nicoleta-Monica Popa-Fotea, Corneliu Iorgulescu, Alexandrina Nastasa, Adelina Pupaza, Viviana Gondos, Ioana-Gabriela Petre, Steliana-Cosmina Paja, Radu-Gabriel Vatasescu

**Affiliations:** 1 Department of Cardiology, Clinic Emergency Hospital of Bucharest, Calea Floreasca 8, 014461 Bucharest, Romania; ralucamitran972@gmail.com (R.-E.M.); fotea.nicoleta@yahoo.com (N.-M.P.-F.); iorgulescu_corneliu@yahoo.com (C.I.); adelina_m_p@yahoo.com (A.P.); i_comanescu@yahoo.com (I.-G.P.); cosmina_steliana.paja@yahoo.com (S.-C.P.); 2 Department IV—Cardio-Thoracic Pathology, Carol Davila University of Medicine and Pharmacy, Eroii Sanitari Bvd. 8, 050474 Bucharest, Romania; 3 Department of Cardiology, “Elias” University Emergency Hospital, 011461 Bucharest, Romania; alexandrina.nastasa@yahoo.com; 4 Department of Medical Electronics and Informatics, Polytechnic University of Bucharest, 060042 Bucharest, Romania; viviana.gondos@hellimed.ro

**Keywords:** left atrial low-voltage areas, left atrial fibrosis, atrial fibrillation, recurrence, radiofrequency ablation

## Abstract

Atrial fibrillation (AF), the most frequently encountered arrhythmia worldwide, is associated with increased cardiovascular morbidity and mortality. Left atrial (LA) and antral region of the pulmonary veins (PVs) remodeling are risk factors for AF perpetuation. Among the methods of LA fibrosis quantification, bipolar voltage mapping during three-dimensional electro-anatomical mapping is less studied. The main aim of this study was to analyze the relationship between the degree of LA fibrosis quantified in low-voltage areas and the efficacy of AF radiofrequency catheter ablation. All consecutive patients with AF ablation were included, and the degree of LA fibrosis was measured based on the low-voltage areas in the LA and the antral region of PVs (<0.5 mV for patients in sinus rhythm and <0.25 mV for patients in AF at the time of the ablation procedure). The efficacy of AF ablation was determined by the rate of recurrence after a blanking period of three months. A total of 106 patients were included; from these, 38 (35.8%) had AF recurrence after RF ablation, while 68 (64.2%) were free of events. The area and percentage of LA fibrosis were significantly higher in the patients with AF recurrence (*p* = 0.018 and *p* = 0.019, respectively). However, no significant differences were found between the patients with and without AF recurrence in terms of the area and percentage of PVs fibrosis (*p* = 0.896 and *p* = 0.888, respectively). Moreover, LA fibrosis parameters proved to be excellent predictors for AF recurrence (areas under the curve of 0.834 and 0.832, respectively, *p* < 0.001) even after adjustment for LA indexed volume and CHA_2_DS_2_-VASc score. In conclusion, LA fibrosis measured on bipolar voltage maps increases the risk of AF recurrence after the RF catheter ablation procedure.

## 1. Introduction

Atrial fibrillation (AF) is the most prevalent arrhythmia in the global population and an important cause of cardiovascular morbidity and mortality [1]. The structural and functional remodeling that occurs in the left atrium (LA) and the antral region of the pulmonary veins (PVs), as a result of AF, contributes to perpetuation, leading to persistent AF (continuously sustained beyond 7 days) and permanent forms of AF (AF that is accepted by the patient and physician, with no further attempts to restore or maintain sinus rhythm) [2,3]. Moreover, it has been demonstrated that alterations in atrial perfusion during AF may lead to LA ischemia and structural remodeling, which further favors the chronicization of AF [4]. Higher pre-procedural, but also postprocedural, fibroses were independently associated with worse outcomes, emphasizing the importance of fibrotic myopathy in the perpetuation of AF substrate [5]. Atrial fibrosis can be quantified by using two-dimensional speckle-tracking echocardiography (2DSTE), late gadolinium enhancement cardiac magnetic resonance imaging (LGE-MRI) and bipolar voltage mapping during three-dimensional 3D electro-anatomical mapping (3D-EAM). Most studies focus on the quantification of the level of fibrosis using 2DSTE by measuring the LA strain and LGE-MRI via the determination of LGE areas. However, few studies focus on the level of fibrosis determined using bipolar voltage mapping by measuring the low-voltage areas. 

Quantifying LA fibrosis using bipolar voltage mapping could be useful to determine the rate of AF recurrence, hypothesizing that the degree of atrial fibrotic tissue could predict the rate of AF recurrence after the radiofrequency catheter ablation procedure. The main aim of the present study was to analyze the relationship between the degree of LA fibrosis quantified as low-voltage areas using LA bipolar voltage mapping and the efficacy of AF radiofrequency catheter ablation.

## 2. Materials and Methods

We present a prospective, observational, two-center (Emergency Clinical Hospital, Bucharest, Romania, and Sanador Clinical Hospital, Bucharest, Romania) study. The patient demographics and clinical characteristics were exported from hospitalization records. All included participants provided written informed consent for the ablation procedure and inclusion in medical research at the time of the procedure.

### 2.1. Study Population

All consecutive patients who underwent radiofrequency catheter ablation procedures for AF between September 2017 and July 2023 were included. Exclusion criterion was the inability to complete LA bipolar voltage maps. Demographic and clinical characteristics (age, associated cardiovascular comorbidities, type of AF-paroxysmal or non-paroxysmal, CHA_2_DS_2_-VASc score), as well as transthoracic echocardiographic (TTE) parameters, were recorded at the time of the procedure. 

### 2.2. Electroanatomic Mapping and Ablation

All procedures were performed under conscious sedation and anesthesia. After the vascular access, a transseptal puncture was performed with the insertion of long sheaths into the LA; a Carto3 mapping system (Biosense Webster, Inc., Irvine, CA, USA) was used for the 3D reconstruction of the LA and 4 pulmonary veins (PVs). The 3D-EAM was accomplished with multielectrode duodecapolar mapping catheters, Lasso^®^ NAV (Biosense Webster) or Pentaray^®^ NAV (Biosense Webster) using an algorithm that allows points acquisition only for the electrodes with good tissue contact. Radiofrequency (RF) catheter ablation was performed with a contact-force-sensing catheter ThermoCool ST/SF (Biosense Webster) using a high-power (50 w/45 C/17 mL/min) short-duration (<20 s) setup guided by the ablation index (400 at the anterior and 350 at the posterior LA wall) [6]. Large antral pulmonary veins isolation (PVI) was performed according to the CLOSE protocol [7] with entrance and exit blocks in all PVs as a procedural end-point. In patients with extensive LA fibrosis, adjunctive posterior wall isolation was performed, with entrance and exit blocks confirmed. For patients who remained in AF at the end of the procedure, electrical conversion to sinus rhythm was performed with authentication of the entrance and exit blocks. All procedures were performed by two expert operators. 

### 2.3. Fibrosis Assessment 

The degree of LA fibrosis was determined by measuring the low-voltage areas at the level of the LA and the antral region of the PVs using bipolar voltage mapping. Low-voltage areas were defined as follows:

- <0.5 mV for patients in sinus rhythm at the time of ablation;
- <0.25 mV for patients in AF at the time of ablation.

A second prerequisite was set to avoid areas of very small voltage signal from being contaminated by noise or insufficient tissue contact; precisely, the presence of voltages less than 0.15 mV was excluded from the analysis. Additionally, two bipolar voltage mapping parameters were measured for each map: area and percentage of fibrosis at the level of the LA and antral region of the PVs. To extend the fibrosis area, the CARTO tool entitled “area measurement” was used, which allows the manual measurement of the color-coded areas by the chosen thresholds presented above, while the fibrosis percentage was determined as the percentage of low voltage from the surface area. The fibrotic percentage categorized each patient into one of the four groups: stage I < 10%, stage II 10–20%, stage III 20–30% and stage IV > 30%, adapted from the Utah classification of atrial fibrosis based on LGE-MRI, according to Marrouche et al. [5]. Due to the manual measurement process, in order to minimize measurement errors, two experienced operators measured the bipolar voltage mapping parameters twice for each patient.

### 2.4. Follow-Up

The efficacy of AF catheter ablation was determined by the rate of AF recurrence after the procedure. A blanking period of 3 months was taken into consideration when establishing the rate of recurrence. All patients underwent control visits 6 and 12 months after the RF procedure with a 12-lead electrocardiogram (ECG) and 24 h Holter monitoring. If patients presented symptoms that indicated AF recurrence, 12-lead ECG and 24 h Holter monitoring were performed earlier. The definition of AF recurrence was considered to be at least one episode of AF or atrial flutter lasting more than 30 s, recorded either on the surface or Holter monitoring. 

### 2.5. Statistical Analysis

The SPSS IBM version 21 (IBM Corp., Armonk, NY, USA) was used for all statistical analyses. The normal distribution of the numerical data was verified using the Kolmogorov–Smirnov test. Categorical variables were expressed in percentages and analyzed using the chi-square test. Numerical variables were presented as mean ± standard deviation and comparisons between two groups were analyzed using Student’s *t*-test if normally distributed or non-parametric tests (Mann–Whitney U-Test) if not. Pearson’s correlation was used to determine the correlation between the two continuous variables. To evaluate the discriminative power of bipolar voltage mapping parameters in AF recurrence, receiver operating characteristic curve (ROC) analysis was performed to determine the area under the curve (AUC), and optimal sensitivity and specificity were calculated. The AUCs were also calculated for composed variables (LA fibrosis area or percentage and LA indexed volume or CHA_2_DS_2_-VASc score) to analyze the possible additive value of these parameters. To evaluate interobserver concordance, the intraclass correlation coefficient (ICC) was applied. A two-sided *p*-value < 0.05 was considered statistically significant for all tests.

## 3. Results

### 3.1. Clinical and Paraclinical Characteristics

A total of 106 patients were included with a mean age of 58.89 ± 10.79 years; for all patient characteristics, see Table 1. At inclusion, there were 91 (85.8%) patients with paroxysmal AF and 15 (14.2%) with persistent AF. Eighty-five (80.2%) subjects were undergoing their first ablation procedure, while 21 (19.8%) underwent ≥1 ablation procedure before. The mean follow-up time was 16.68 ± 13.99 months (between 4 and 70 months). After RF catheter ablation for AF, 38 (35.8%) patients had AF recurrence, while 68 (64.2%) patients were free of events. 

Subjects with AF recurrence had significantly larger indexed LA volumes (44.4 ± 14.53 mL/m^2^, *p* = 0.005) as well as more reduced LVEF (52.28 ± 9.95%, *p* = 0.047) compared with those without AF recurrence (35.82 ± 10.28 mL/m^2^, and 55.62 ± 5.24%, respectively), but did not present any other significant differences regarding other echocardiographic parameters.

### 3.2. Electroanatomic Mapping and Low-Voltage Area Study

**Figure 1:**
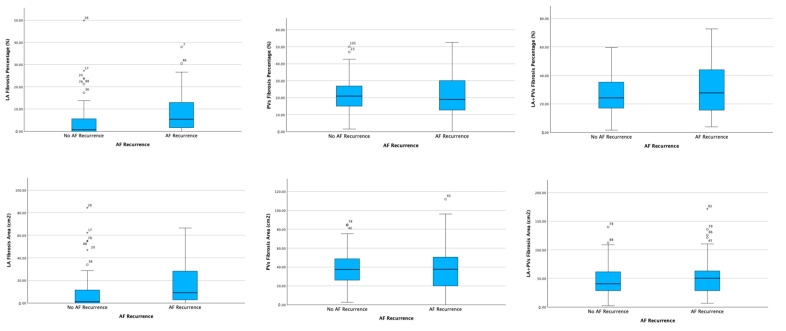
Bipolar voltage mapping parameters: comparison between maps in no atrial fibrillation (AF) and AF recurrence groups. LA, left atrium; PVs, pulmonary veins.

Although the areas of LA fibrosis were significantly smaller in patients with no AF recurrence (9.06 ± 16.95 cm^2^) compared with those with AF recurrence (17.82 ± 19.9 cm^2^, *p* = 0.018), as well as the percentage of LA fibrosis (4.62 ± 8.55% versus 8.94 ± 9.72%, *p* = 0.019), there were no statistically significant differences in the areas (38.72 ± 18.75 cm^2^ versus 39.29 ± 25.63 cm^2^, *p* = 0.896) and percentages (21.58 ± 10.29% versus 21.26 ± 12.95%, *p* = 0.888) of PVs fibrosis between the two groups (Table 2, Figure 1). Moreover, patients free from AF after ablation had longer procedures (3076.30 ± 1608.40 s versus 2936.97 ± 1592.42 s, *p* = 0.684), but lower total radiofrequency application time (1047.42 ± 479.55 s versus 1093.88 ± 554.26 s, *p* = 0.667) compared with patients who relapsed, although these results were not statistically significant (*p* > 0.05) (Table 2).

**Figure 2:**
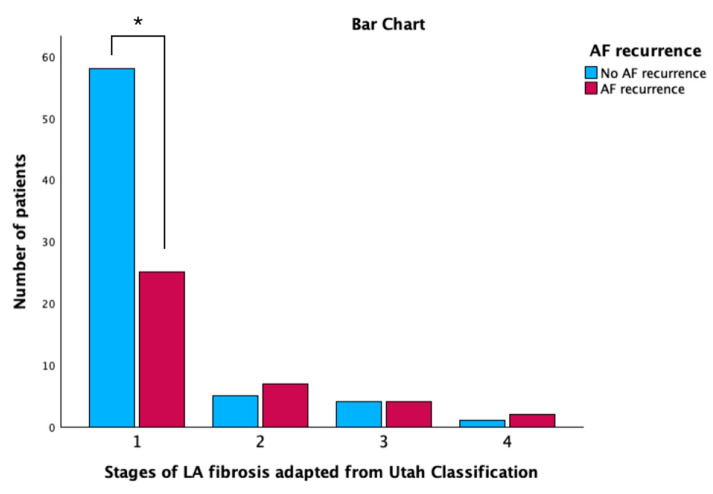
Stages of left atrium fibrosis adapted from Utah Classification between the group with and without atrial fibrillation recurrence. * *p* < 0.05. LA left atrium; AF atrial fibrillation.

**Figure 3:**
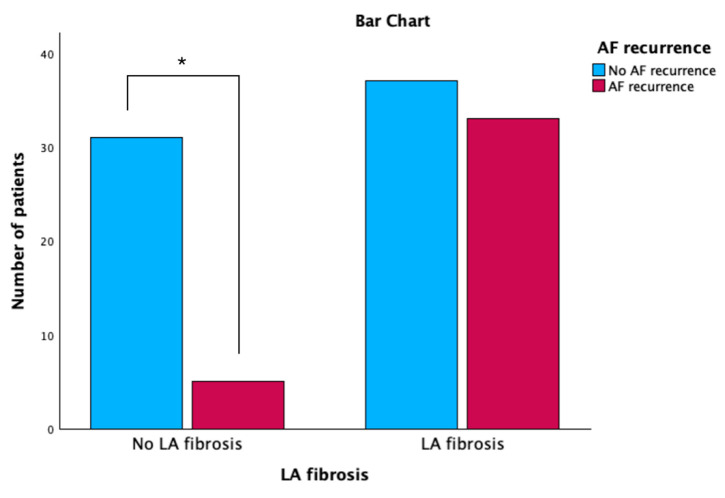
Bar chart graph showing the relationship between the presence of LA fibrosis and AF recurrence after the ablation procedure for atrial fibrillation. * *p* < 0.05. LA, left atrium; AF, atrial fibrillation.

According to LA fibrosis adapted from the Utah classification [5], there are significantly fewer patients in stage 1 in the group with AF recurrence compared with the group without AF recurrence (*p* < 0.001) (Figure 2). Furthermore, the association between the presence of LA fibrosis and AF recurrence after radiofrequency catheter ablation was statistically significant (*p* < 0.001) (Figure 3). 

**Figure 4:**
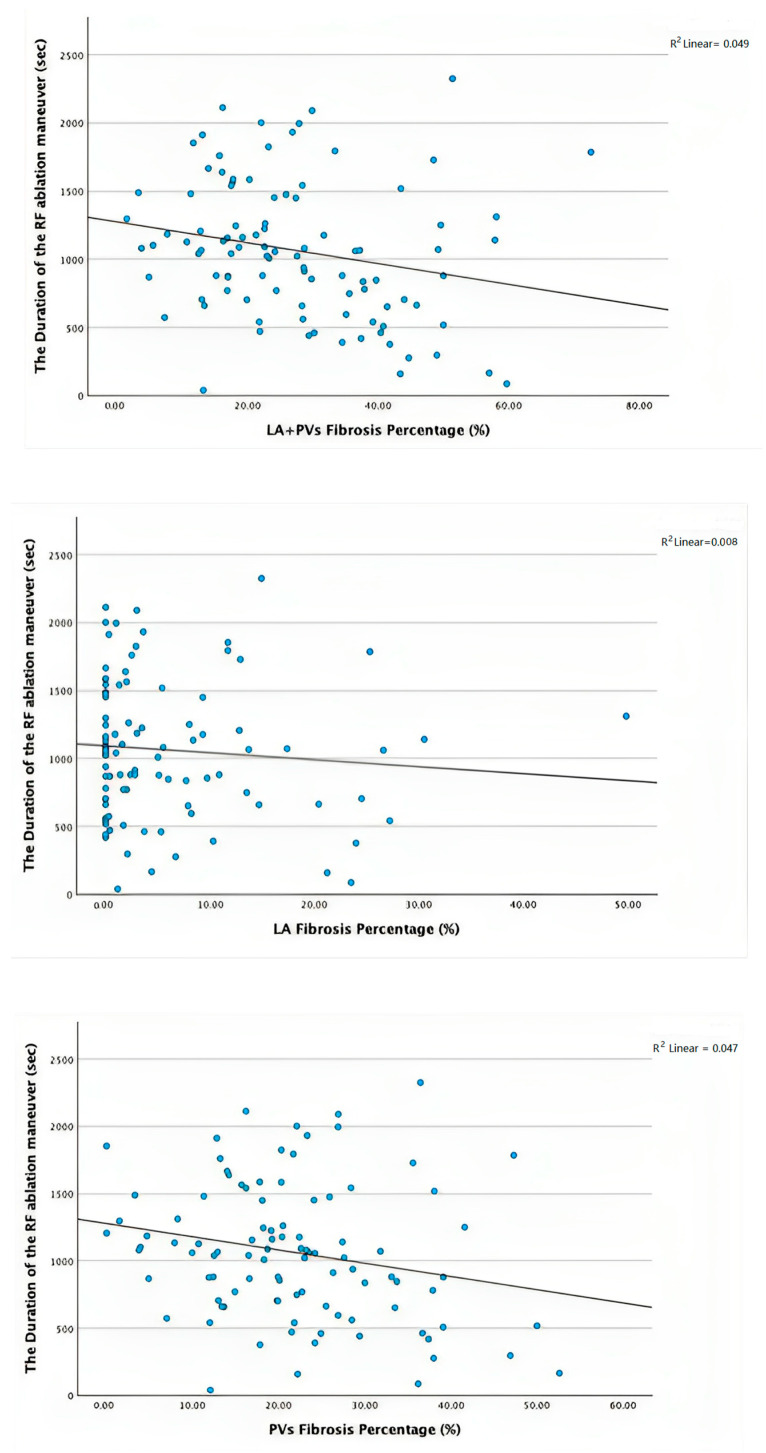
Scatterplot graphs which show the correlation between total RF application time and low-voltage areas (LA + PVs, PVs, LA). RF, radiofrequency; LA, left atrium; PVs, pulmonary veins.

A significant correlation was found between RF duration and the total percentage of low-voltage areas (LA + PVs) (*p* = 0.028, r = −0.221) as well as between total RF application time and the percentage of PVs fibrosis (*p* = 0.03, r = −0.218). However, there was no significant correlation between the total RF application time and the percentage of LA low-voltage areas (*p* = 0.377, r = −0.090) (Figure 4).

**Figure 5:**
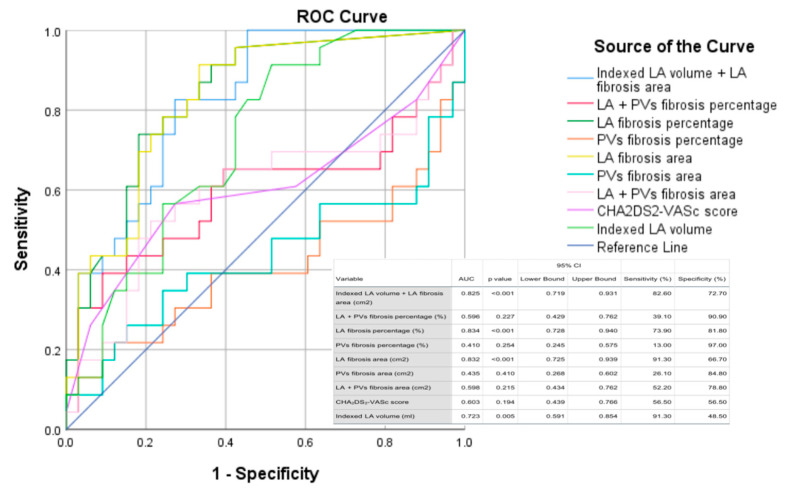
ROC curve for bipolar voltage mapping parameters. LA, left atrium; PVs, pulmonary veins.

According to the ROC curves among all bipolar voltage mapping parameters, the percentage and area of LA fibrosis showed to be the best predictors for AF recurrence with an area under the curve (AUC) of 0.834 and 0.832, respectively (Figure 5). LA indexed volumes and CHA_2_DS_2_-VASc scores had smaller AUCs, and the composed variable (LA indexed volume and LA fibrosis area) did not significantly improve the accuracy of the predictions. 

### 3.3. Reproducibility Assessment 

The ICC was calculated based on the measurements of the two independent observers for each of the included patients. The results showed adequate correlations with ICC between 0.72 and 0.84 for all bipolar voltage mapping parameters.

## 4. Discussion

Left atrial fibrosis as a hallmark of atrial myopathy can be quantified using either one of the three following methods. The first method uses 2DSTE to measure the LA strain. Several studies [8,9,10,11] have investigated the role of the left atrial strain in predicting the rate of AF recurrence after the ablation procedure and found that global and lateral strains of the LA were independent predictors of AF recurrence. Therefore, the LA strain could be a useful indicator for patient selection in AF RF catheter ablation [8]. The second method uses LGE-MRI to measure the areas in the LA that presented LGE [5]. The DECAAF study conducted by Marrouche et al. concluded that atrial fibrosis estimated by this MRI is independently related to the probability of AF recurrence after the ablation procedure [5]. The third method, employed in the present study, uses low-voltage areas measured using bipolar voltage mapping to determine LA fibrosis. As an advantage, this approach does not require additional investigations or catheters and does not expose patients to additional risks. It is demonstrated that LA low-voltage areas measured using bipolar voltage mapping correlate with LA fibrosis [12]. Therefore, these low-voltage areas have been a target for extensive AF catheter ablation in the hope of improving ablation outcomes, especially in patients with non-paroxysmal AF who usually present a less favorable ablation outcome [12]. However, randomized controlled trials and observational studies show mixed results, so there is not enough evidence to conclude that voltage-guided ablation improves procedure outcomes [12]. 

The present study shows that there is a significant association between the presence of LA fibrosis measured using bipolar voltage mapping and AF recurrence after RF catheter ablation. The degree of LA fibrosis measured using bipolar voltage maps was categorized into four classes adapted from the Utah classification [5]. We used only the quantitative aspect of the grading because there are no unequivocal studies that demonstrate a significant correlation between the degree of LA fibrosis measured using LGE-MRI and electro-anatomical mapping [13]. Moreover, the degree of LA fibrosis was significantly lower in patients who were AF-free after the ablation procedure than in subjects presenting with recurrent episodes of AF. Notwithstanding, no significant dissimilarity was found regarding the number of low-voltage zones at the antral regions of the pulmonary veins, an element that would be expected, given that the current standard in AF RF ablation targets only the isolation of the PVs. Furthermore, the percentage and area of LA fibrosis showed to be the best predictors of AF recurrence; precisely, the LA fibrosis percentage demonstrated high sensitivity (73.9%) and specificity (81.8%) for a cutoff of ≤3.3%, as did the area of LA fibrosis with 91.3% sensitivity and 66.7% specificity for a cutoff of ≤1.25 cm^2^. The addition of LA indexed volume or CHA_2_DS_2_-VASc score to the LA fibrosis parameters did not improve the accuracy of predicting AF recurrence. Interestingly, we found an inverse correlation between the RF duration and the total percentage of low-voltage areas, as well as between the RF duration and the percentage of PV fibrosis in patients with AF recurrence. This can be the result of the ablation protocol that utilized high-power, short-duration RF applications inducing wider area and less deep lesions with a higher chance of a gapless ablation line [14], but also due to the anatomical and ultrastructural characteristics of the atrial myocytes compared with their ventricular counterparts. This is probably because the atria are thin-walled chambers and the entrance and exit blocks are easier to obtain after the myocardium is already affected, although there are no studies on the histopathological characterization of RF ablation in atrial scars, compared with the existing data on ventricular myocardium [15]. The LA fibrotic degeneration increases the arrhythmogenic substrate affecting the electrical conduction and the duration of an action potential, increasing the re-entrant activity and thus the development of non-PV AF [16]. Indeed, the areas of LA fibrosis measured using bipolar voltage mapping were significantly larger in patients with AF recurrence. Considering that most of the ablation procedures in this study population were PVI alone, the LA low-voltage areas suggest that the existence of extra PV arrhythmogenic substrates could predict which patients would benefit more from extending the RF ablation in these areas of LA fibrosis to reduce the rate of recurrence.

## 5. Limitations

It is warranted to mention several limitations of the study. First, the small cohort of patients included may have produced false-positive results or overestimated the magnitude of an association. Second, the inclusion of redo-ablations may have induced bias due to prior ablations, which could have affected the AF substrate. Third, the lack of data regarding the LA LGE or LA global and regional strain; LA changes measured by 2DSTE, precisely, in the transport function were associated with AF occurrence and could have influenced the AF burden in our cohort of patients [17]. Fourth, the short period of follow-up raises the possibility that recurrent AF episodes might have been missed. Fifth, the cutoff values for low-voltage areas are variable in the literature and may have influenced the calculation of the bipolar voltage maps.

## 6. Conclusions

In conclusion, the area and percentage of LA fibrosis measured using bipolar voltage mapping were significantly higher in patients with AF recurrence. Moreover, the area and percentage of LA fibrosis were excellent predictors of AF recurrence after AF radiofrequency catheter ablation. However, these findings did not apply to the percentage and area of PV fibrosis. Therefore, these results suggest the need to improve the LA-specific management of AF ablation procedures.

## Figures and Tables

**Table 1:** Clinical characteristics of the study population (*n* = 106).

Variable	Value
Age (years)	58.89 ± 10.79
Sex (male), *n* (%)	61 (57.54%)
Smoking, *n* (%)	16 (15.1%)
Hypertension, *n* (%)	66 (62.3%)
Obesity, *n* (%)	32 (30.2%)
Diabetes mellitus, *n* (%)	16 (15.09%)
Obstructive sleep apnea syndrome, *n* (%)	41 (38.68%)
Dyslipidemia, *n* (%)	77 (72.64%)
Coronary Artery Disease, *n* (%)	10 (9.4%)
Congestive Heart Failure, *n* (%)	45 (42.45%)
CHA_2_DS_2_-VASc score at inclusion, *n* (%)	
0	20 (18.9%)
1	26 (24.5%)
2	22 (20.8%)
3	24 (22.6%)
4	11 (10.4%)
5	3 (2.8%)

**Table 2:** The bipolar voltage mapping parameters in patients with or without AF recurrence.

Bipolar Voltage Mapping Parameter	Patients without AF Recurrence (*n* = 68)	Patients with AF Recurrence (*n* = 38)	*p*-Value
Area of LA fibrosis (cm^2^)	9.06 ± 16.95	17.82 ± 19.9	0.018
Percentage of LA fibrosis (%)	4.62 ± 8.55	8.94 ± 9.72	0.019
Area of PVs fibrosis (cm^2^)	38.72 ± 18.75	39.29 ± 25.63	0.896
Percentage of PVs fibrosis (%)	21.58 ± 10.29	21.26 ± 12.95	0.888
Total area (LA + PVs) of fibrosis (cm^2^)	47.78 ± 27.71	57.11 ± 37.57	0.148
Total percentage (LV + PVs) of fibrosis (%)	26.20 ± 13.20	30.20 ± 16.47	0.175
Total RF application time (s)	1047.42 ± 479.55	1093.88 ± 554.26	0.667
Total Ablation Time (s)	3076.30 ± 1608.40	2936.97 ± 1592.42	0.684

LA left atrium; PVs pulmonary veins; RF radiofrequency.

## Data Availability

Data are available upon request from the corresponding author.
